# Book Reviews

**Published:** 1899-08

**Authors:** 


HOLDEN'S HUMAN OSTEOLOGY. Comprising a description of the bones, with delineations of the attachments of the muscles, the general and microscopic structure of bone and its development. Edited by CHARLES STEWART, F. R. S., Conservator of the Museum of the Royal College of Surgeons of England; and R. W. REID, M. D., F. R. C. S., Regius Professor of Anatomy in the University of Aberdeen. Octavo, pp. ix.—358. Eighth edition, illustrated. Philadelphia: P. Blakiston's Son & Co. 1899.

Every student of anatomy must look with delight upon this work. A study of osteology has been usually considered dry; but Holden has demonstrated that there really "is poetry in bones." The descriptive text makes the subject so plain and it is written in a way to give it such a charm that there is really no further excuse for ignorance of the bony anatomy of the human subject.

The first thirty-six pages of the work are descriptive of bone structure and its general composition, including its microscopical study and the transformation of cartilage into bone. These various branches of the subject are presented with absorbing interest and an accuracy of detail that is beyond criticism.

In his description of the several bones of the human body Holden surpasses any anatomist with whom we are familiar. Nearly one-third of the book is devoted to a description of the skull alone and no student of the subject can examine these pages without great admiration for the manner in which the bones of the skull have been described or without praise for the systematic manner in which the author has done his work.

Holden's description of the pelvis is another marvel in the way of teaching bone anatomy and his entire description of the human skeleton is simply admirable. In his comparative osteology Holden demonstrates his knowledge in paleozoology, and it is a fascinating addition to the usual way of presenting bone anatomy to the student.

The text is supported all the way through with accurate drawings on stone, the majority of which were originally made from nature by the author. A more superb piece of book-making has not been seen in many a day. With Gerrish's anatomy and Holden's osteology the text-books of human anatomy are complete.

TRANSACTIONS OF THE MEDICAL SOCIETY OF THE STATE OF NORTH CAROLINA. Forty-fifth Annual Meeting, held at Charlotte, May 3, 4 and 5, 1898. Winston: Carolina Publishing Co. 1898.

This volume comes to us this year bound in cloth, which is a decided improvement over former editions, and gives the work of this admirable society a stability that it deserves. Besides the president's address it is the custom of this society to persent an annual oration and an annual essay. In this instance these are presented by president Francis Duffy, of Newbern; Dr. Albert Anderson, of Wilson; and Dr. W. C. Brownson, of Asheville, respectively. The remainder of the volume is filled with original contributions and reports of committees, including the report of the state board of health.

---

HAY-FEVER AND ITS SUCCESSFUL TREATMENT. By W. C. HOLLOPETER, A. M., M. D., Clinical Professor of Pediatrics in the Medico-Chirurgical College of Philadelphia; Physician to the Methodist-Episcopal Hospital, etc. Second edition, revised and illustrated. Philadelphia: P. Blakiston's Son & Co. 1899.

The demand for the second edition of this little monograph within a year from its first publication is both a compliment to the author and to the discriminating audience which he addressed. Hay-fever is a perennial dread to its victims and has been until recently a bête-noir to physicians. This author states that he has given complete relief to more than 200 patients in his private practice; he has made a thorough clinical study of the disease and an exhaustive review of the literature relative to it. These facts entitle his treatise to patient consideration, as well as to the confidence of the profession of medicine. We commend it to all interested, whether physicians or patients.

---

TRANSACTIONS OF THE AMERICAN ASSOCIATION OF OBSTETRICIANS AND GYNECOLOGISTS. Eleventh Annual Meeting, held at Pittsburg, September 20, 21 and 22, 1898. Edited by WILLIAM WARREN POTTER, M. D., Secretary. Philadelphia: Wm. J. Dornan, Printer. 1899.

This volume presents a group of papers upon important subjects relating to obstetrics, gynecology and abdominal surgery. The president, Dr. Charles A. L. Reed, chose for the subject of his address, The evolution of specialism in medicine. It is a scholarly paper, abounding in classic lore, and is one of the most entertaining presidential addresses of the year. We think the whole profession of medicine might derive beneficial information from its careful reading. Another paper out of the common is Dr. Price's essay on The professional nurse and her training. Every nurse ought to possess a copy of this paper. Dr. Henry Howitt read a most valuable contribution relating to The surgical treatment of intussusception of infants. Dr. Howitt's remarkable experience is shown by the fact that he has seen seven cases of this disease in children under one year, four of which have recovered after abdominal section for its relief. Dr. Cumston's paper on Septic infection of ovarian cystoma is a most valuable contribution to the literature of this subject. Every paper in the book presents practical aspects for the considera-tion of gynecologists, obstetricians and abdominal surgeons. The discussions make them stand out in strong light.

---

Report of the Commissioner of Education for the Year 1896-97. Volume II. Containing Parts II. and III. Washington: Government Printing Office. 1898.

In this volume among other things is a chapter entitled, The learned professions and social control. It is written by Mr. Well-ford Addis, specialist in the bureau of education, and contains much of interest to doctors and lawyers. A table, in two columns, gives the essentials required by the several states for entrance to these two professions, and there are several engravings of laboratories in foreign universities.

Another chapter gives statistics of professional schools that may be consulted with interest and profit by all concerned.

---

Progressive Medicine, Vol. II. A Quarterly Digest of Advances, Discoveries and Improvements in the Medical and Surgical Sciences. Edited by Hobart Amory Hare, M. D., Professor of Therapeutics and Materia Medica in the Jefferson Medical College of Philadelphia. Octavo, cloth, 472 pages, fifty-six illustrations and three full-page plates. Philadelphia and New York: Lea Brothers & Co.

The second volume of this quarterly presents carefully prepared and exhaustive papers upon the following subjects: Surgery of the abdomen, including hernia, by William B. Coley, M. D., of New York; Gynecology, by John G. Clark, M. D., of Philadelphia; Diseases of the blood, diathetic and metabolic disorders, diseases of the spleen, thyroid gland and lymphatic system, by Alfred Sten-gel, M. D., of Philadelphia; Ophthalmology, by Edward Jackson, M. D., of Denver.

These subjects are full of practical thought for the busy physi-cian and they have been presented in a manner that will command attention. Our opinion unreservedly expressed as to the first vol-ume holds equally good in its application to this one. The experience of the editor stands for much in the creation of such a book, and Dr. Hare has amply demonstrated in more fields than one his high scientific and literary ability.

The literary pendulum is swinging toward the quarterly standard. Such periodicals appear frequently enough to keep pace with the progress of medicine and they enable writers to furnish carefully prepared material in studied form, the result of mature thought and in a fashion that will endure. We trust that a high standard of illustration, already established in the first and second volumes, will continue through the series. Good illustrations serve to accentuate the text and their free employment is to be stimulated whenever and wherever practicable.

---

INTERNATIONAL CLINICS. A Quarterly of Clinical Lectures on Medicine, Neurology, Surgery, Gynecology and Obstetrics, Ophthalmology, Laryngology, Pharyngology, Rhinology, Otology and Dermatology, and Specially Prepared Articles on Treatment and Drugs. By professors and lecturers in the leading medical colleges of the United States, Germany, Austria, France, Great Britain and Canada. Edited by JUDSON DALAND, M. D. (Univ. of Penna.), Philadelphia, Instructor in Clinical Medicine and Lecturer on Physical Diagnosis in the University of Pennsylvania; Assistant Physician to the Hospital of the University of Pennsylvania, etc.; Fellow of the College of Physicians of Philadelphia. Volume I. Ninth series. 1899. Octavo, pp. x.—303. Philadelphia: J. B. Lippincott Co. 1899.

The prominent lectures in this volume are Cold as an antipyretic, by Horatio C. Wood; Treatment of uterine fibromas, by A. Ricard; Injuries to the head. by John Ashurst, Jr.; Rational view of the appendicitis question, by John B. Roberts; Treatment of burns, by William G. Dugan; Use of electricity in surgery, by Alexander J. C. Skene; Extensive carcinoma of the lower lip, and resection of the superior maxilla for carcinoma, by James H. Dunn, and Lupus vulgaris, by Edmond Lesser. The entire contents, however, deals with subjects of interest many of which are well illustrated.

---

TWENTIETH CENTURY PRACTICE. An International Encyclopedia of Modern Medical Science. By leading authorities of Europe and America. Edited by THOMAS L. STEDMAN, M. D., New York City. In twenty volumes. Vol. XVI., "Infectious Diseases." New York: William Wood & Co. 1899.

The first article in this volume treats of Lobar pneumonia and is written by Andrew H. Smith, of New York. It is especially strong in pathology and Smith takes the view that it is an infectious disease. Under the head of etiology he states that while the essential cause of pneumonia is the development of a specific germ in the pulmonary alveoli, it is evident that, as a rule, there must be a contributing cause that in some way lays the system open to attack. In other words, that in any given case there is a condition present that favors either the migration of the germ into the lung or its development when there, or both. As to its communicability, he says that while pneumonia is unquestionably a communicable disease, it is not readily communicable from person to person. We have quoted substantially the author's words because they represent the most modern opinion on these points. The entire article should be read to be appreciated.

The next monograph is on Cerebrospinal meningitis, by A. Netter, of Paris. The importance of this subject is such as to justify a careful study of this contribution to its literature. The third title is Dysentery, by A. A. DeAzevedo Sodré, of Rio de Janeiro. The literature of the subject has been carefully collated and its pathology well considered. Yaws forms the subject of the next article, which is written by H. A. Alford Nicholls, Dominica, W. I.; Ernst Ziegler, of Freiburg, I. B., contributes an article on Inflammation; Otto G. T. Kiliani, of New York, writes on Erysipelas; Landon B. Edwards, of Richmond, Va., describes simple continued fever; Leo Popoff, of St. Petersburg, discourses on Relapsing fever; John S. Thatcher and John Winters Brannan, of New York, conclude the book with exhaustive articles on Typhoid fever.

Considered by and large, this is one of the most interesting and important volumes of the series for the general practitioner.

---

Defective Eyesight. The Principles of Its Relief by Glasses. By D. B. St. John Roosa, M. D., LL.D., Professor Emeritus of Diseases of the Eye, New York Post-Graduate Medical School and Hospital; Surgeon to the Manhattan Eye and Ear Hospital, etc. Duodecimo, pp. x.—193. Illustrated. New York: The Macmillan Co. 1899.

This practical little volume, published formerly under another title, has been completely rewritten by the distinguished author, teacher, clinician and practitioner, who not only adorns the special fields of his professional work, but is an ornament to the whole domain of medicine.

This publication in its new form and as it now stands will become one of the greatest aids to the general physician, who is most frequently first consulted in regard to defective eyesight. It instructs him how to proceed early in the simpler cases. There are so many symptoms that may come from eyestrain and other defects of vision, that it becomes the family doctor to be alert as to their quick detection and real significance. He should be competent at least to give the proper advice as to the seeking of counsel.

Refractive errors are nowadays quite common and muscle errors often contribute to the discomfort of patients. These must needs get speedy relief of the most intelligent kind, in order that the symptoms they produce may be permanently relieved. In this book will be found full consideration of these subjects. The value of the ophthalmometer is fully set forth as an aid to the diagnosis of many diseases and conditions, but there is very little said in regard to the employment of mydriatics.

It is pleasant to know that, whereas formerly ophthalmologists were prone to resort to the aid offered by these agents, now it is almost exceptional for a reputable ophthalmologist to use mydriatics. The science has so progressed that they are very rarely needed.

The printing of the book makes it easy reading, the type and color of paper rendering it pleasant to the eye.

The profession owes to the distinguished president of the Post-graduate Medical School and Hospital a debt of gratitude for the writing of this work, and it should be in the hands of every one who makes any pretense to the practice of medicine.

Atlas of External Diseases of the Eye. By Dr. O. Haab, of Zurich. Edited by G. E. de Schweinitz, M. D., Professor of Ophthalmology, Jefferson Medical College of Philadelphia. With 100 colored illustrations. Philadelphia: W. B. Saunders. 1899.

Saunders's medical hand atlases are getting recognition everywhere as a part of the best medical literature of the period. Time was when the general physician knew very little of diseases of the eye, except possibly the acute inflammations of its structures. Now, with the rapid and accurate dissemination of knowledge relating to many of the more intricate yet common affections of the visual organ, any and every physician, no matter what his specialty, is expected to possess a reasonable degree of information upon at least the common diseases. This remark applies especially to refractive errors and muscular faults that occasion a train of unpleasant reflexes —symptoms that often deceive the novice and perplex the expert.

This book will aid the average practitioner of medicine to reach an intelligent conclusion as to conditions existing and the remedy for the same, in so far as they relate to external diseases of the eye. These are those that demand immediate action, oftentimes, to save sight and unless taken in the right way, even then vision may be lost or seriously impaired. The illustrations are so plain and the descriptive text is so clear, that it would be difficult to err, either in diagnosis or treatment, if Haab is closely studied and carefully followed as a guide. It is very difficult to portray in colors the diseases herein considered, yet the author has succeeded in doing so with amazing accuracy. The American editor has increased the value of the book for an American audience by the smooth yet accurate translation he has made, as well as by the comments he has occasionally interpolated in brackets. The name of de Schweinitz is an additional guarantee, if one were needed, of the value of this work.

